# Optical See-Through Head-Mounted Displays With Short Focal Distance: Conditions for Mitigating Parallax-Related Registration Error

**DOI:** 10.3389/frobt.2020.572001

**Published:** 2020-12-04

**Authors:** Fabrizio Cutolo, Nadia Cattari, Umberto Fontana, Vincenzo Ferrari

**Affiliations:** ^1^Information Engineering Department, University of Pisa, Pisa, Italy; ^2^Department of Translational Research and New Technologies in Medicine and Surgery, EndoCAS Center, University of Pisa, Pisa, Italy

**Keywords:** augmented reality, optical see-through displays, registration, calibration, parallax related error

## Abstract

Optical see-through (OST) augmented reality head-mounted displays are quickly emerging as a key asset in several application fields but their ability to profitably assist high precision activities in the peripersonal space is still sub-optimal due to the calibration procedure required to properly model the user's viewpoint through the see-through display. In this work, we demonstrate the beneficial impact, on the parallax-related AR misregistration, of the use of optical see-through displays whose optical engines collimate the computer-generated image at a depth close to the fixation point of the user in the peripersonal space. To estimate the projection parameters of the OST display for a generic viewpoint position, our strategy relies on a dedicated parameterization of the virtual rendering camera based on a calibration routine that exploits photogrammetry techniques. We model the registration error due to the viewpoint shift and we validate it on an OST display with short focal distance. The results of the tests demonstrate that with our strategy the parallax-related registration error is submillimetric provided that the scene under observation stays within a suitable view volume that falls in a ±10 cm depth range around the focal plane of the display. This finding will pave the way to the development of new multi-focal models of OST HMDs specifically conceived to aid high-precision manual tasks in the peripersonal space.

## 1. Introduction

The overarching goal of any augmented reality (AR) display is to seamlessly enrich the visual perception of the physical world with computer-generated elements that appear to spatially coexist with it. This aspect, that can be referred to as locational realism (Grubert et al., 2018), is the main factor that provides the user with a sense of perceptual consistency. Wearable AR head-mounted displays (HMDs) ideally represent the most ergonomic and reliable solutions to support complex manual tasks since they preserve the user's egocentric viewpoint (Sielhorst et al., 2006; Vávra et al., 2017; Cutolo et al., 2020).

In optical see-through (OST) HMDs the direct view of the world is mostly preserved and there is no perspective conversion in viewpoint and field of view (fov), as with video see-through (VST) systems. This aspect confers a clear advantage over VST solutions, particularly when used to interact with objects in the peripersonal space, since it allows the user to maintain an unaltered and almost natural visual experience of the surrounding world (Rolland and Fuchs, 2000; Cattari et al., 2019). This aspect is critical for instance in highly challenging manual tasks as in image-guided surgery, where reality preservation and fail-safety are essential features (van Krevelen and Poelman, 2010; Qian et al., 2017b).

OST displays use semi-transparent surfaces (i.e., optical combiners) to optically combine the computer-generated content with the real view of the world (Holliman et al., 2011). The virtual content is rendered on a two-dimensional (2D) microdisplay placed outside the user's fov and collimation lenses are placed between the microdisplay and the optical combiner to focus the virtual 2D image so that it appears at a pre-defined and comfortable viewing distance on a virtual image plane (i.e., the display focal plane) (Rolland and Cakmakci, 2005).

Nowadays, optical see-through (OST) HMDs are at the cutting edge of the AR research, and several consumer level headsets have been recently developed following the success of the Microsoft HoloLens 1 (e.g., MagicLeap One, HoloLens 2, Meta Two, Avegant, Lumus DK Vision). Despite this surge in consumer access, the successful use of these devices in practical applications is still limited by the complexity and unreliability of the calibration procedures needed to ensure an accurate spatial alignment between real-world view and computer-generated elements rendered onto the see-through display (Qian et al., 2017a; Cutolo, 2019). The inaccessibility of the user-perceived reality, makes indeed OST display calibration particularly challenging (Gilson et al., 2008).

The calibration aims to estimate the intrinsic and extrinsic parameters of the virtual rendering camera (Grubert et al., 2018). These parameters account for the eye position with respect to the OST display and encapsulate the projection properties of the eye-NED pinhole model.

State-of-the art manual (Genc et al., 2002; Tuceryan et al., 2002; Navab et al., 2004; Moser and Swan, 2016) or interaction-free (Itoh and Klinker, 2014a,b; Plopski et al., 2015) OST display calibration procedures either partially or fully rely on user interaction and provide sub-optimal results that are not tolerable for those high-precision applications for which the accurate alignment between virtual content and perceived reality is of the utmost importance. Moreover, this process should be theoretically repeated whenever the HMD moves and causes a change in the relative position between the virtual image plane of the OST display and the user's eye (i.e., center of projection of the virtual rendering camera). This would entail re-estimating the position of the eye's first nodal point (i.e., the center of projection of the user's eye) with respect to the OST display. Unfortunately, manual calibration procedures are tedious and error-prone, whereas interaction-free methods based on eye-tracking devices are only capable of indirectly estimating the center of rotation of the user's eye(s) and not the actual center(s) of projection, which is usually shifted by 7–8 mm (Guestrin and Eizenman, 2006). Furthermore, the pose of the eye-tracker with respect to the display might change during use because the user may unintentionally move the camera or the camera needs to be re-oriented to be adjusted for different users and eye positions. As a result, frequent re-calibrations of the camera would be required. For these reasons, none of these approaches are capable to completely remove the virtual-to-real registration error due to the viewpoint shift (i.e., parallax). Overall, if the parallax between the calibrated rendering camera and the actual user's viewpoint remains uncorrected, the virtual-to-real registration error will grow with the depth difference between the virtual image plane of the display and the observed scene (Luo et al., 2005). Unfortunately, the focal plane of most consumer-level OST NEDs is at infinity or at a distance that is incompatible with its use as an aid to manual activities (as it is far from the peripersonal space) (Ferrari et al., 2020).

Indeed, owing to the uncertainty in the calibration of the viewpoint-dependent rendering camera, the Microsoft HoloLens can have a maximum static registration error of < 10 mrad, which results in an error of about 5 mm at a distance of 50 cm from the user. This value of the registration error was experimentally verified by Condino et al. (2018) in their study.

To counter this problem, in this work we present a strategy that significantly mitigates the registration error due to the viewpoint shift around a pre-defined depth in the user's peripersonal space. Our solution follows the intuition by Owen et al. (2004), and demonstrates the beneficial impact, on the virtual-to-real registration, of the adoption of OST displays whose optical engines collimate the computer-generated image at a depth close to the fixation point of the user at close distances. This feature, if coupled with a single camera-based calibration procedure performed for a generic viewpoint, is capable to substantially mitigate the AR registration error due to the viewpoint shift for working areas around the depth of the focal plane of the OST display. We also demonstrate that, with this solution, there is no need for any prior-to-use calibration refinement, either manual or interaction-free, to maintain the accurate virtual-to-real registration provided that the view volume under observation stays within a suitable depth range around the optical depth of the display image. This finding will pave the way to the development of new multi-focal models of OST HMDs specifically conceived as aid during high-precision manual tasks in the peripersonal space.

## 2. Geometry of the Optical See-Through Displays and Perspective Projection Equations

### 2.1. Notation

The following notation is used throughout the paper. Lower-case letters represent scalars. Coordinate systems are denoted by uppercase letters (e.g., the rendering camera coordinate system associated with the calibration point R_C_). The origin of any reference systems is denoted by uppercase bold letters (e.g., the origin of the rendering camera coordinate system **C**). Points/vectors are denoted by lowercase bold letters with a superscript indicating the reference coordinate system (e.g., a 3D point in the world reference system **p**^W^). Matrices are denoted by uppercase typewriter letters, such as the intrinsic matrix of the off-axis rendering camera Koff−E. Rigid transformation matrices are denoted by uppercase typewriter letters with subscript and superscript representing the source and destination reference frames respectively (e.g., the rigid transformation between W and R_*C*_ is $\left[{\;}_{\text{W}}^{{\text{R}}_{C}}\text{R}\;{\;}_{\text{W}}^{{\text{R}}_{C}}\text{t}\right]$).

### 2.2. Pinhole Camera Model

**Figure 1:**
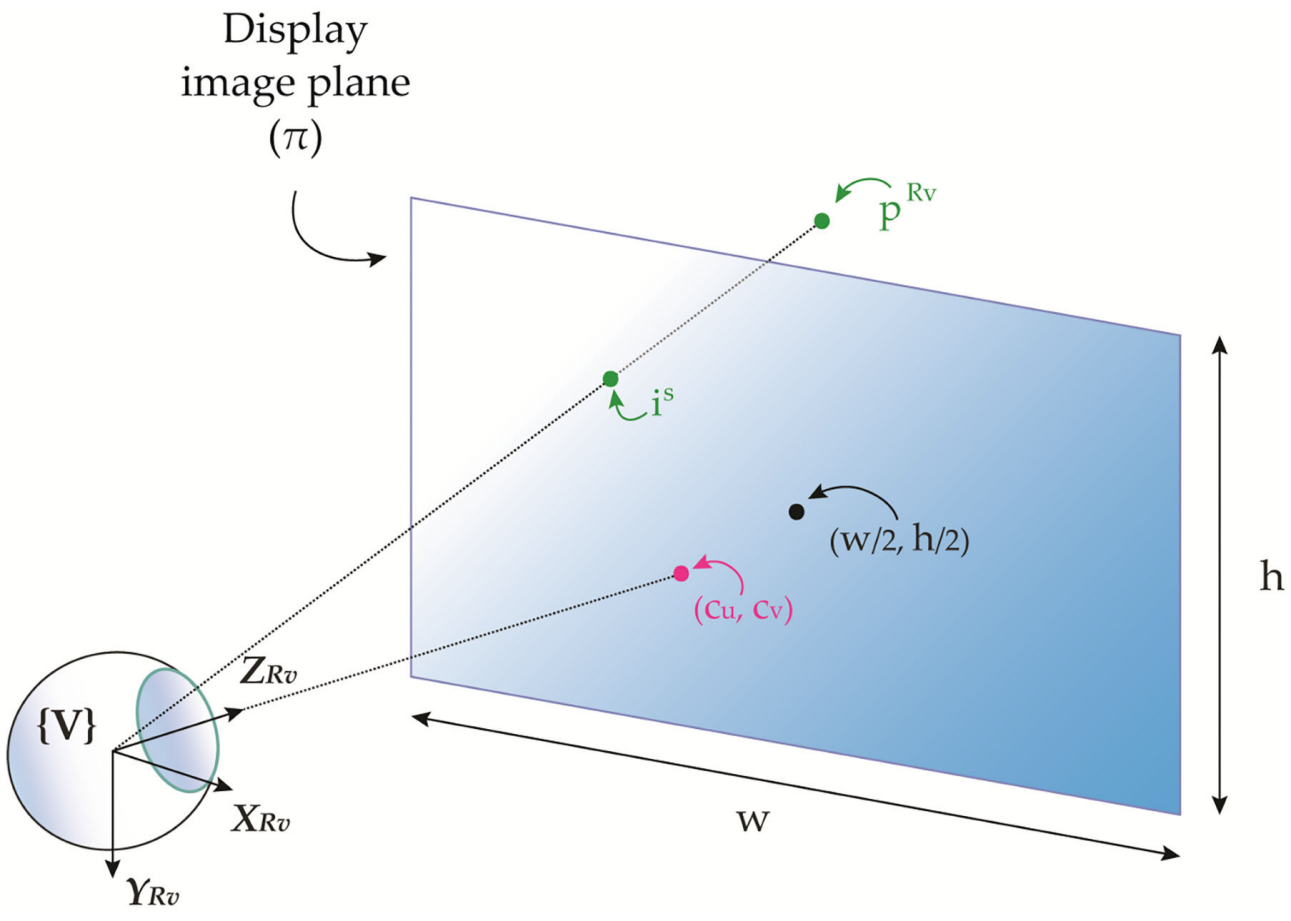
3D representation of the combined eye-display off-axis pinhole model comprising the eye as the projection center and the see-through virtual screen as the image plane.

The combined eye-display system of an OST display is commonly modeled as an off-axis pinhole camera where the nodal point of the user's eye corresponds to the center of projection **V** and the see-through virtual screen of the display corresponds to the image plane S (Figure 1). The intrinsic matrix of the off-axis pinhole camera model of the eye-display system for a generic position of the eye is:


(1) Koff−E=[fu0cu0fvcv001]

where *f*_*u*_ and *f*_*v*_ are the focal lengths of the display in pixels, denoting the distances from the image plane S to the pinhole camera projection center **V**. Notably, the focal lengths are different for non-perfectly square pixels for which the pixel aspect ratio is not 1. The principal point is defined as the intersection between the principal axis of the see-through display and the display image plane. The pixel coordinates of the principal point are (*c*_*u*_, *c*_*v*_).

This model represents the perspective projection transformation of the virtual rendering camera that maps a random point in the 3D rendering camera space ${\text{p}}^{{\text{R}}_{\text{V}}}$ to the associated 2D pixel displayed on the image plane **i**^S^ of the OST display.


(2) λiS=Koff−E[I3×3  03×1]pRV

where both points are expressed in homogeneous coordinates, and λ is a generic scale factor due to the equivalence between points in homogeneous coordinates.

The above formulation, assumes a special choice of the world reference system W, with W ≡ R_V_. The 3 × 4 general projection transformation that maps world points onto the image plane of the display P encapsulates also the extrinsic parameters (i.e., the 6DoF rigid transformation from W to R_V_):


(3) λiS=Koff−E[RWRV   tWRV] pW=(P)RVpW

Since λ is arbitrary, Equation (3) is an up-to-scale relation and the independent parameters to be computed are 11. Therefore, any calibration of OST display, aims to calculate the 11 independent projection parameters of the virtual rendering camera ( P)_R_V__ that generates the correct mapping of each 3D vertex of the virtual object onto the image plane of the OST display. This is commonly done by solving for all of the matrix components at once, or by systematically determining the parameters in Equation (3).

### 2.3. Camera-Based OST Calibration Method With Homography Correction

**Figure 2:**
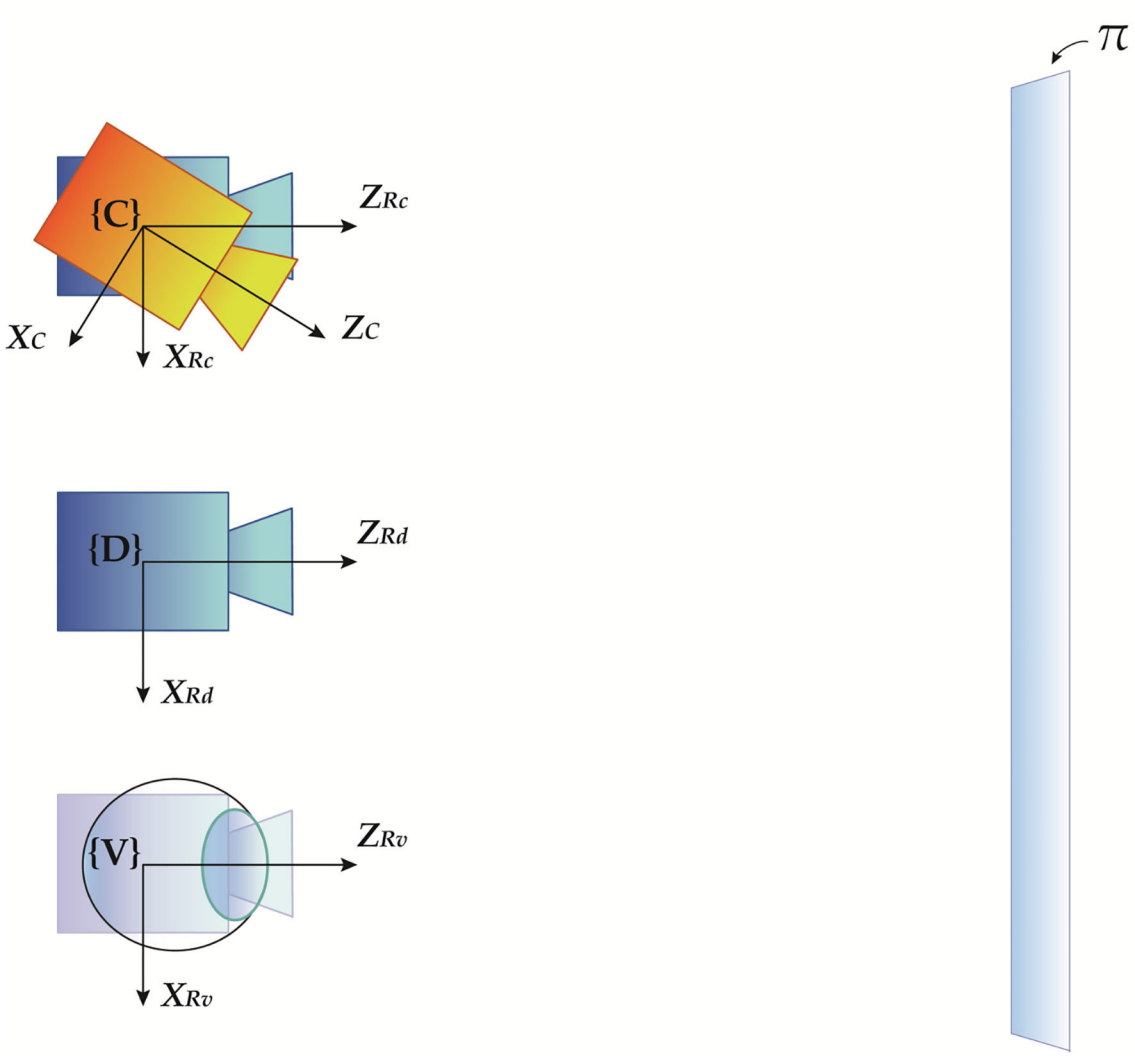
Geometrical representation of the spatial relationships between the four reference systems involved in the camera-based calibration procedure, illustrating the relevant coordinate systems: the ideal on-axis virtual rendering camera R_D_, the real off-axis rendering camera associated with a generic calibration position R_C_, the physical viewpoint camera used as a replacement of the user's eye C, and the off-axis rendering camera associated with the real user's eye position. Rendering cameras are black-colored, whereas the physical viewpoint camera is orange-colored.

In a previous work (Cutolo et al., 2019), we presented an off-line camera-based calibration method for OST displays. We refer to Cutolo et al. (2019) for more details on the method. Here we report the key equations underpinning the calibration procedure as they are the starting points for our analysis of the registration error. The method exploits standard camera calibration and photogrammetry techniques to produce the off-axis camera model of the eye-display system Koff−E for a generic viewpoint position **C**. Hereafter, this position will be referred to as the calibration position. Figure 2 shows a schematic diagram illustrating the spatial relationships between all the reference systems involved in the calibration procedure as well as in the overall perspective projection. Similarly the same relationships are listed in Table 1.

**Table 1:** Position and orientation of all the reference systems involved in the OST perspective projection relation.

**Reference systems**	**Position**	**Orientation**
On-axis rendering camera R_D_	Center of the display eye-box *D*	Orientation of the display image plane
Off-axis rendering camera (at calibration position) R_C_	Calibration position *C*	Orientation of the display image plane
Physical viewpoint camera C	Calibration position *C*	Orientation of the viewpoint camera image plane
Off-axis rendering camera (at viewpoint position) R_V_	User's viewpoint position *V*	Orientation of the display image plane

As described in more detail in Cutolo et al. (2019), the perspective projection equation of the off-axis rendering camera associated with the calibration point is obtained using the induced-by-a-plane homography relation between the points on the viewpoint camera image plane and those on the OST display image plane:


(4) λiS=Kon-E(RCRD+tCRD(nC)⊺dC→π)[ WCR   tWC] pW       =Kon-E(I3×3+tCRD(nRD)⊺dC→π)RCRD[RCRD    tWC]pW

The same relation in matrix form is:


(5) λ(iS)C=Kon-E×HC︸(Koff-E)C×RCRD[RWC   tWC]︸TextpW              =Kon-E×HC︸(Koff-E)C×[RWRD  RWRD  tWC︷tWRC]︸TextpW

Therefore, the matrix Koff−E can be computed by applying a planar homography correction ${\text{H}}_{\text{C}}$ to the intrinsic matrix of the ideal on-axis model of the rendering camera that models the eye-display system Kon−E. This latter is determined by using the manufacturer's specifications of the OST display, and it is ideally located at the center of the eye-box of the display **V**, where the eye-box consists of the range of allowed eye's positions, at a pre-established eye-to-combiner (i.e., the eye-relief) distance, from where the whole image produced by the display is visible. This homography correction encapsulates the shift and scaling effect due to a particular viewpoint position, and it also accounts for the deviations of the real optical features of the see-through display from the ones provided by the manufacturer's specifications.

The method relies on a camera used as a replacement of the user's eye and placed within the eye-box of the OST display (i.e., at the calibration position **C**). This camera is referred to as the viewpoint camera.

To compute the homography correction ${\text{H}}_{\text{C}}$ and the rotation between display and camera image plane ${\;}_{\text{ C}}^{{\text{R}}_{\text{D}}}\text{R}$ , a virtual checkerboard pattern is displayed on the image plane of the OST display and observed by the viewpoint camera. Hence, by solving a standard P*n*P problem, we can calculate the relative pose between the viewpoint camera C reference system and the ideal on-axis rendering camera $\left[{\;}_{\text{ C}}^{{\text{R}}_{\text{D}}}\text{R}\;{\;}_{\text{ C}}^{{\text{R}}_{\text{D}}}\text{t}\right]$.

Notably, all the rendering cameras have the same orientation (i.e., the orientation of the display) and therefore we have ${\;}_{\text{ C}}^{{\text{R}}_{\text{D}}}\text{R}\equiv {\;}_{\text{C}}^{{\text{R}}_{\text{C}}}\text{R}$; this latter relation explains how all the rotational transformations between any viewpoint-dependent rendering camera reference system (${\;}_{\text{C}}^{{\text{R}}_{\text{C}}}\text{R}\;\forall \;\text{C}$) and the reference system of the physical viewpoint camera (C) are the same. On account of this, Equations (5) and (3) are equivalent provided that the user's eye position (viewpoint) matches the calibration position V≡C. Further, the rotational contribution of ${\text{T}}_{\text{ext}}$ (i.e.,${\;}_{\text{W}}^{{\text{R}}_{\text{D}}}\text{R}$) in Equation (5) is the same regardless of the orientation of the viewpoint camera used for the calibration as it represents the relative orientation between world reference system and display.

The translation vector ${\;}_{\text{ C}}^{{\text{R}}_{\text{D}}}\text{t}$ is used to compute ${\text{H}}_{\text{C}}$ and it provides us a measure of the shifting and scaling contribution due to the viewpoint shift from the ideal on-axis location of the rendering camera to the real viewpoint location (i.e., the calibration position where the viewpoint camera is located).

The homography correction accounting for the viewpoint position and the real optical features of the display is:


(6) HC=[dC→πdR→π0tCRD xdR→π0dC→πdR→πtCRD ydR→π001]

where *d*^**C**→π^ is the distance from the calibration point **C** and the virtual image plane of the OST display π (i.e., the display focal plane), and *d*^**R**→π^ is the distance from the ideal center of projection of the on-axis rendering camera **R** and π. The planar homography ${\text{H}}_{\text{C}}$ encapsulates the shift and scaling effect due to the measured translation vector ${\;}_{\text{ C}}^{{\text{R}}_{\text{D}}}\text{t}$ that is induced by the particular viewpoint position with respect to the ideal on-axis position of the rendering camera.

### 2.4. Viewpoint Shift Contribution to the Eye-Display Pinhole Model

In a real application, the actual viewpoint position is different from the calibration position **V**≠**C**. Therefore, the intrinsic matrix should be further refined by applying an additional homography correction encapsulating the shift and scaling effect associated with the relative translation from the calibration position and the current viewpoint position (Itoh and Klinker, 2014b; Cutolo et al., 2019):


(7) (Koff-E)V=(Koff-E)C[1+z′dC→π0−x′dC→π01+z′dC→π−y′dC→π001]                       =(Koff-E)C×HC→V

where ${\;}_{{\text{R}}_{\text{C}}}^{{\text{R}}_{\text{V}}}\text{t}={\;}_{\text{C}}^{\text{V}}\text{t}=\left[x^{\prime },y^{\prime },z^{\prime }\right]$ is the translation vector from the calibration point to the current viewpoint position. As anticipated, all off-axis rendering camera systems associated with different viewpoint positions share the same orientation (i.e., the orientation of the image plane of the display). Thus, the rotation correction ${\;}_{{\text{R}}_{\text{C}}}^{{\text{R}}_{\text{V}}}\text{R}=I_{3x3}$.

By plugging Equation (7) in Equation (5), we have:


(8) λ(iS)V=(Koff-E)C×HC→V×[I  tCV]×Text pw

Therefore, the viewpoint shift ${\;}_{\text{C}}^{\text{V}}\text{t}$ generates two parallax contributions to the projection relation of the eye-display model: an intrinsic contribution, represented by the homography correction ${\text{H}}_{\text{C→V}}$, and an extrinsic contribution represented by $\left[I\;{\;}_{\text{C}}^{\text{V}}\text{t}\right]$. The accurate real-to-virtual registration is maintained only if both these two contributions are accurately estimated by tracking the viewpoint (e.g., with an eye-tracking mechanism).

## 3. Conditions for Mitigating Parallax-Related Registration Error

Overall, the viewpoint shift, if not compensated for through an intrinsic and extrinsic correction of the perspective projection relation, generates a registration error. Nonetheless, there are points in the space for which this error is theoretically null regardless of the amount of viewpoint shift. These are the points belonging to the image plane of the see-through display, for which ${\text{p}}_{z}^{\text{w}}=d^{\text{C}\to \pi }$. Without loss in generality, let us assume that *W* ≡ *C*. Equation (8) becomes:


(9) λ(iS)V=(Koff-E)C×HC→V×[I  tCV] ×[pxRC pyRC dC→π]T

A parallax between calibration position and viewpoint position tCV=[x′ y′ z′]T generates the following contributions:


(10) $${\text{H}}_{C\to \text{V}}=\left[\begin{array}{ccc} 1+\frac{z^{\prime }}{d^{C\to \pi }} & 0 & \frac{-x^{\prime }}{d^{C\to \pi }}\\ 0 & 1+\frac{z^{\prime }}{d^{C\to \pi }} & \frac{-y^{\prime }}{d^{C\to \pi }}\\ 0 & 0 & 1 \end{array}\right]$$

and


(11) [I  tCV=[I  [x′ y′ z′]T]

Therefore, Equation (9) becomes:


(12) λ(iS)V=(Koff-E)C×[dV→πdC→π0−x′dC→πx′0dV→πdC→π−y′dC→πy′001z′][ pxRCpyRCdC→π1]

**Figure 3:**
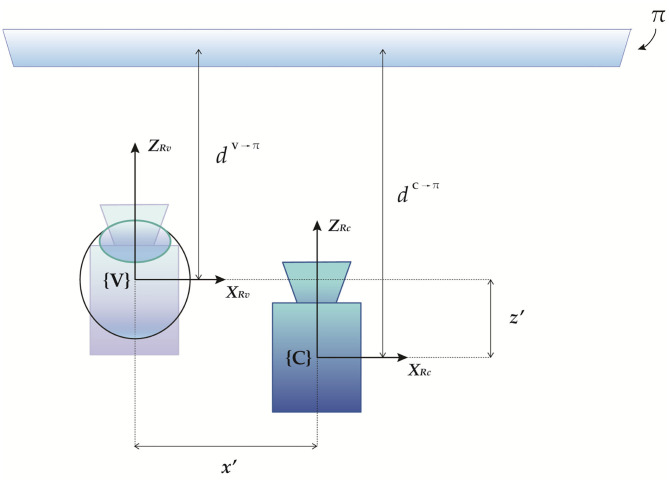
Geometrical representation of the parallax between the calibration position and the viewpoint position.

Geometrically, it is easy to demonstrate the following relation: *d*^**V**→π^ = *d*^**C**→π^ + *z*′ (Figure 3). Therefore, we obtain:


(13) λ(iS)V=(Koff-E)C×[dV→πdC→π pxRCdV→πdC→πpyRCdV→π]

Then, by normalizing by *d*^**V**→π^, we obtain the equivalence of the image point location observed from the two different viewpoints **C** and **V**:


(14) λ(iS)V=(Koff-E)C×[dV→πdC→πpxRCdV→πdC→πpyRCdV→π]~(Koff-E)C×[pxRCdC→πpyRCdC→π1]                 =λ(iS)C

**Figure 4:**
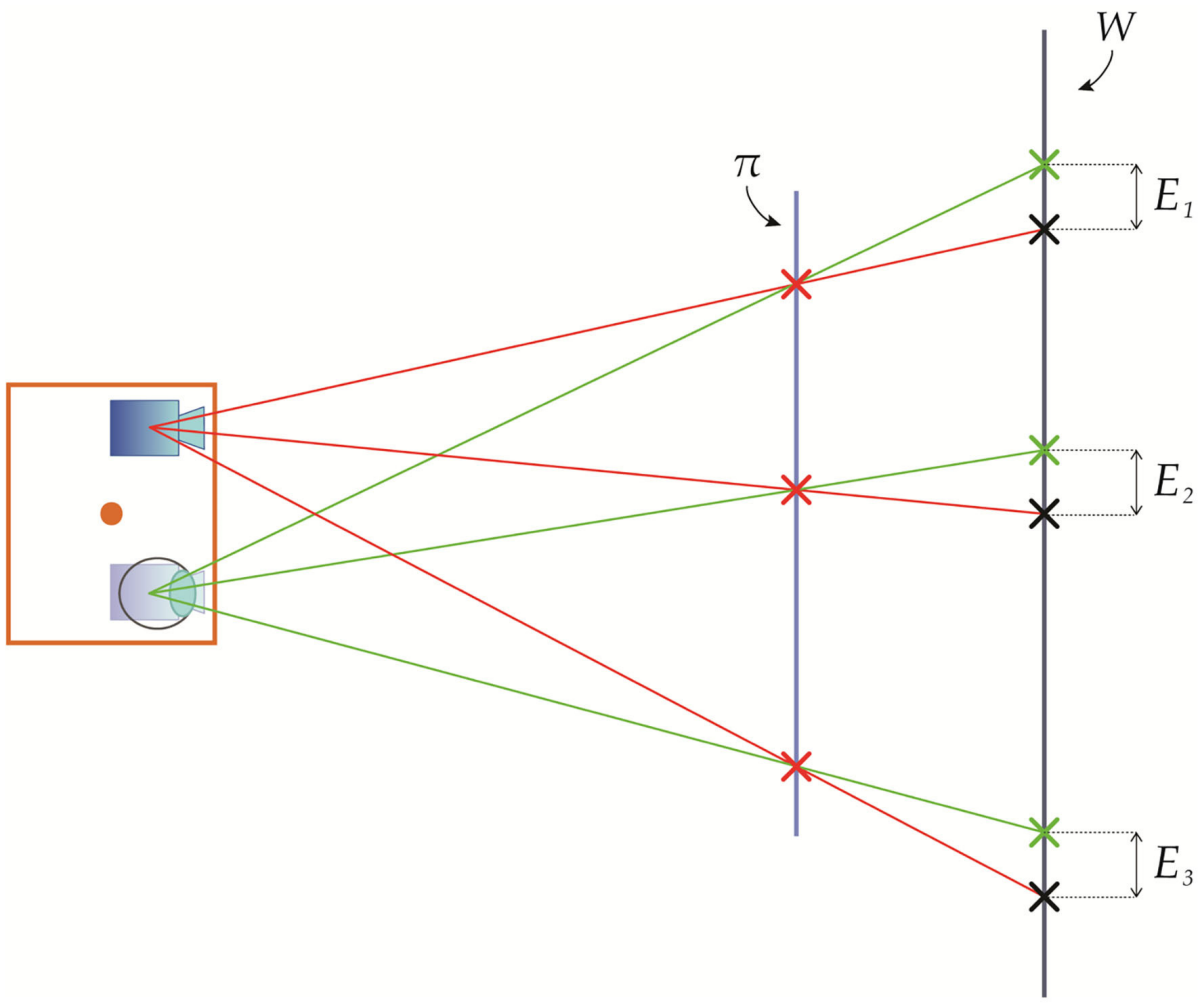
Geometrical representation of the registration error due to the viewpoint shift between a viewpoint position and a calibration position for world points located exactly at the image plane and outside the image plane of the OST display.

Thus, Equation (20) implies that the parallax-related registration error (i.e., due to the viewpoint shift) is null for those world points located exactly at the image plane of the OST display (Figure 4). On the other hand, for the points in space with ${\text{p}}_{z}^{{\text{R}}_{\text{C}}}\neq d^{\text{C}\to \pi }$, the viewpoint shift generates a registration error of:


(15) λ(iS)V=(Koff-E)C×[dV→πdC→π pxRC−x′(pzRCdC→π−1)dV→πdC→π pyRC−y′(pzRCdC→π−1)pzRC+z′]

From simple algebraic manipulations, the registration error due to the viewpoint shift is (in vector form and Euclidean coordinates):


(16) $$\begin{array}{l} E=\frac{p_{z}^{{\text{R}}_{\text{C}}}+z^{\prime }}{f}((i^{\text{S}}{)}_{V}-(i^{\text{S}}{)}_{C})\\ \;=\left[\begin{array}{l} \frac{p_{x}^{{\text{R}}_{\text{C}}}\;z^{\prime }}{p_{z}^{{\text{R}}_{\text{C}}}}(\frac{p_{z}^{{\text{R}}_{\text{C}}}}{d^{C\to \pi }}-1)-x^{\prime }(\frac{p_{z}^{{\text{R}}_{\text{C}}}}{d^{C\to \pi }}-1)\\ \frac{p_{y}^{{\text{R}}_{\text{C}}}\;z^{\prime }}{p_{z}^{{\text{R}}_{\text{C}}}}(\frac{p_{z}^{{\text{R}}_{\text{C}}}}{d^{C\to \pi }}-1)-y^{\prime }(\frac{p_{z}^{{\text{R}}_{\text{C}}}}{d^{C\to \pi }}-1) \end{array}\right] \end{array}$$

**Figure 5:**
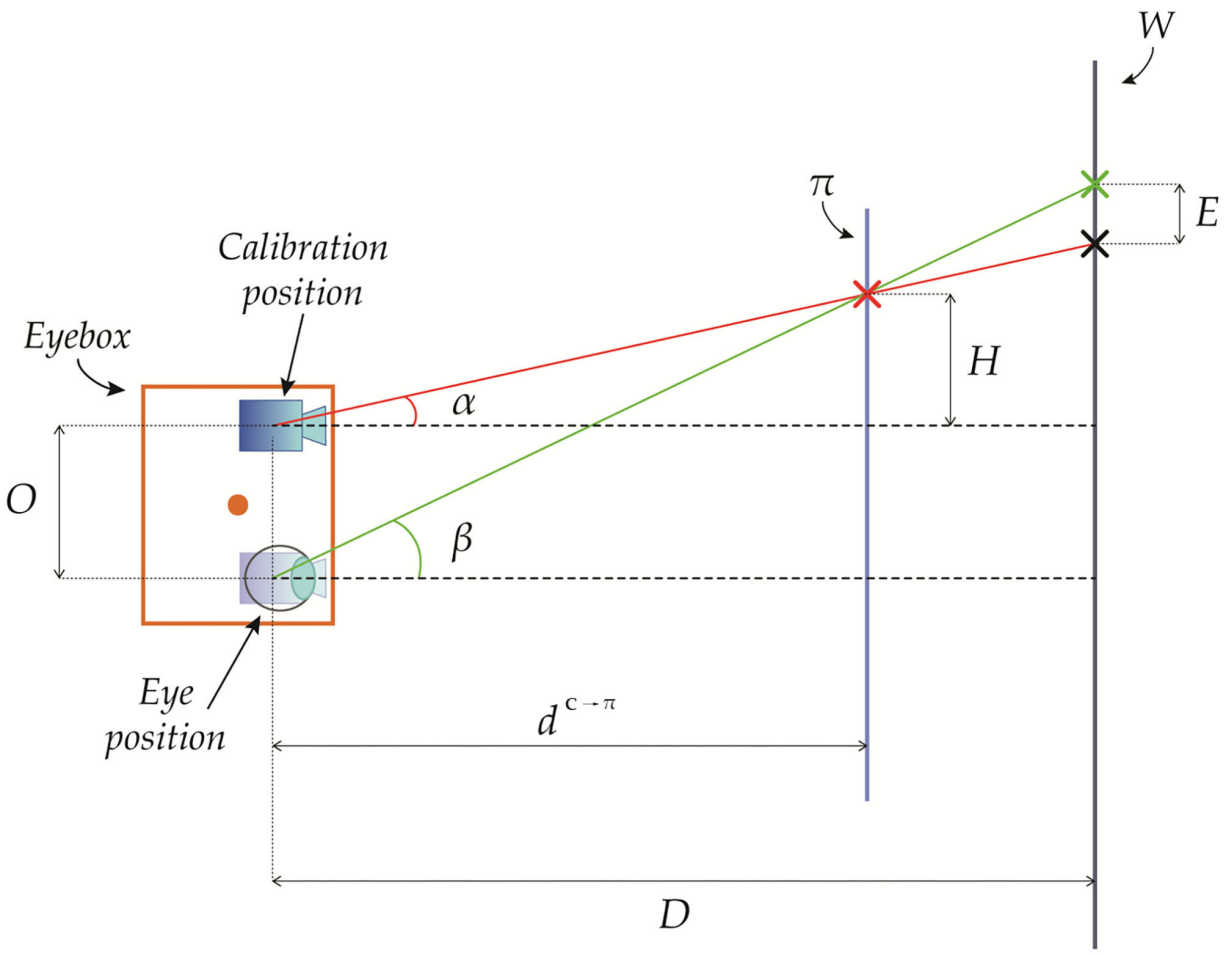
Geometrical representation of the registration error due to a viewpoint shift between a viewpoint position and a calibration position at a depth ${\text{p}}_{z}^{{\text{R}}_{\text{C}}}>d^{\text{C}\to \pi }$.

In Figure 5, we provide a geometrical representation of the registration error due to a viewpoint shift along the x-axis (${\;}_{C}^{V}\text{t}=\left[x^{\prime }\;0\;0\right]$).

## 4. Experimental Setting and Calibration Procedure

**Figure 6:**
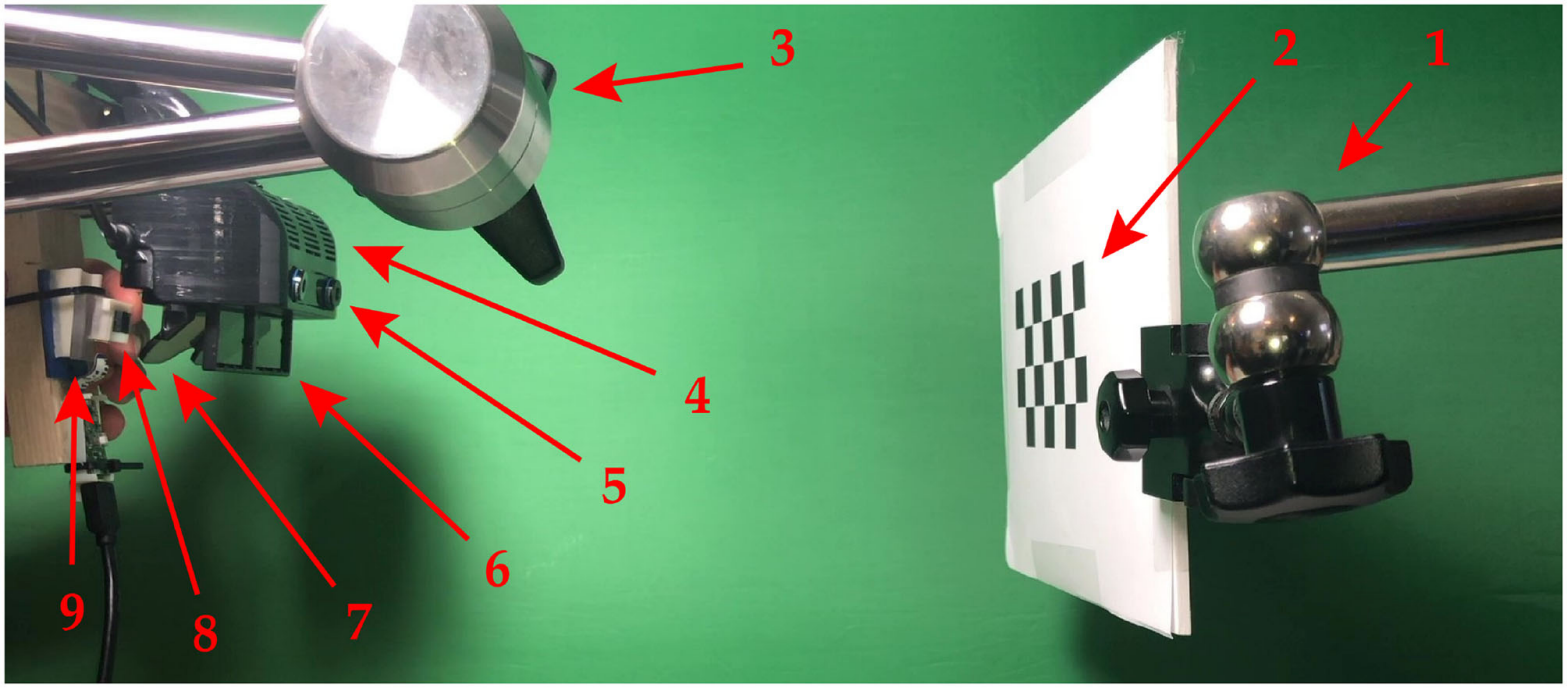
Experimental setting for the calibration procedure and the experimental session. 1→ The validation checkerboard holder. 2→ The validation checkerboard. 3→ The optical see-through head mounted display (OST HMD) holder. 4→ The the OST HMD. 5→ The tracking camera. 6→ The optical shutter. 7→ The optical combiner. 8→ The viewpoint camera. 9→ The 3D printed mounting template.

The Figure 6 shows the experimental setup. In our tests, we used a commercial OST HMD (the ARS30 by Trivisio, Luxemburg) duly customized. The ARS30 visor is provided with a pair of 1280x1024 OLED microdisplays and a pair of optical engines that collimate the computer-generated image to a depth compatible with manual tasks in the peripersonal space. Each microdisplay has a 30° diagonal angle of view resulting in an average angular resolution of ≈ 1.11 arcmin/pixel, and an eye-box dimension of about 8x10 mm. In our experiments, we used only the right display of the sHMD.

With an approach similar to the one proposed by Cutolo et al. (2017, 2020), we housed the HMD in a 3D printed plastic shell whose function is to incorporate a pair of liquid crystal optical shutters that allowed us to occlude the see-through view upon request and remove the real-world background. As viewpoint camera, we used a SONY FCB-MA130, which has a 1/2.45″ CMOS sensor, a 1280x720 resolution, a 59° diagonal FOV, and an angular resolution of ≈ 2.67 arcmin/pixel. The HMD was also integrated with a USB camera placed above the display (Leopard Imaging LI-OV580) for the inside-out tracking mechanism; this camera supports M12 lenses: in our tests, we used a 2.8 mm lens (f-number f2.0) that, associated with a camera resolution of 1280x720, results in a 109° diagonal angle of view. The focal distance of the display *d*^**V**→π^ was empirically measured using the same camera equipped with a lens having a 17.5 mm focal length, a f-number f5.6, and a circle-of-confusion size of 0.025 mm. This particular lens was associated with a narrower field-of-view compared to the 2.8 mm lens and to a wider depth-of-field. Therefore, by measuring the depth-of-field of the camera when the display was in focus, we were able to estimate the value of *d*^**C**→π^ ≈ 33.5 cm. The calibration procedure was performed as follows. First, the viewpoint camera and the tracking camera were both calibrated with a conventional calibration technique (Zhang, 2000) that requires storing multiple camera views of a planar pattern (i.e., OpenCV checkerboard). The linear parameters (i.e., intrinsic camera matrix) and non-linearities due to the camera lens distortion were computed using non-linear least-squares minimization (i.e., Levenberg–Marquardt algorithm). This procedure was performed using the MATLAB camera calibration toolbox (R2019b MathWorks, Inc., Natick, MA, USA).

Next, the viewpoint camera was placed at the calibration point **C**, empirically and approximately set in the center of the eye-box of the display and at the eye relief distance (i.e., ≈ 30 mm from the optical combiner). As done in Owen et al. (2004), this was done by moving the viewpoint camera left and right so as to determine the width of the viewable area of the display and then averaging the extents to determine the center. The same process was performed to establish also the vertical position.

A standard 7x4 OpenCV checkerboard with a square size of 20 mm was used as the target object to be tracked; hereafter this board will be referred to as the validation checkerboard.

The viewpoint-dependent elements of Equation (8) were determined as follows. The composite extrinsic transformation matrix ${\text{T}}_{\text{ext}}$ was estimated by means of the tracking camera whose coordinate system is L.

${\text{T}}_{\text{ext}}$ can be broken down into two main components:

- $\left[{\;}_{\text{W}}^{\text{L}}\text{R}\;{\;}_{\text{W}}^{\text{L}}\text{t}\right]$, which represents the pose of the world reference system with respect to the tracking camera reference system.
- ${\;}_{\text{ C}}^{{\text{R}}_{\text{D}}}\text{R}\left[{\;}_{\text{L}}^{\text{C}}\text{R}\;{\;}_{\text{L}}^{\text{C}}\text{t}\right]=\left[{\;}_{\text{L}}^{{\text{R}}_{\text{D}}}\text{R}\;{\;}_{\text{ C}}^{{\text{R}}_{\text{D}}}\text{R}{\;}_{\text{L}}^{\text{C}}\text{t}\right]$, which represents the rigid transformation between the tracking camera and the off-axis rendering camera located at the calibration point.

$\left[{\;}_{\text{W}}^{\text{L}}\text{R}\;{\;}_{\text{W}}^{\text{L}}\text{t}\right]$ was determined by localizing the validation checkerboard in front of the see-through display, whereas $\left[{\;}_{\text{L}}^{\text{C}}\text{R}\;{\;}_{\text{L}}^{\text{C}}\text{t}\right]$ was determined through a standard stereo calibration routine implemented in OpenCV (OpenCV API version 3.3.1).

As anticipated, and as done in Cutolo et al. (2019), both the rotational contribution caused by the different orientation of the rendering camera with respect to the viewpoint camera ${\;}_{\text{ C}}^{{\text{R}}_{\text{D}}}\text{R}$, and the translation vector ${\;}_{\text{ C}}^{{\text{R}}_{\text{D}}}\text{t}$ that allows us to compute the homography correction accounting for the viewpoint position ${\text{H}}_{\text{C}}$, were estimated by rendering a virtual structured marker of known size on the see-through display and by localizing its inner corners through **C**. This calibration routine was developed in MATLAB environment and using the Computer Vision Toolbox.

## 5. Experiments and Results

### 5.1. Test Design

A dedicated AR application implemented in MATLAB was used to measure the virtual-to-real overlay accuracy, namely the registration error (Figure 8A). In the routine, we generated a virtual scene that consisted of a set of virtual spots observed by a virtual viewpoint (i.e., the rendering camera) whose intrinsic and extrinsic projection parameters were initialized according to the calibration procedure previously described. In this way, for each viewpoint position, we were able to measure the virtual-to-real registration accuracy in terms of Euclidean distance between real landmarks (the corners of the validation checkerboard) and virtual features (the virtual dots).

**Figure 7:**
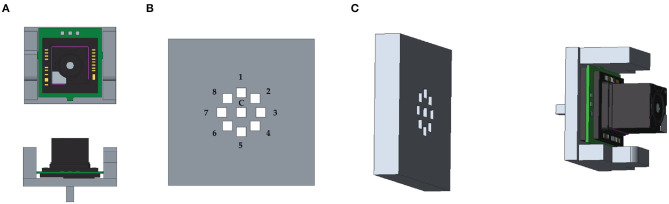
**(A)** 3D CAD of the mounting template used for placing the viewpoint camera in the calibration position (*c*) and in the eight different viewpoint positions within the eye-box of the see-through display. **(B)** The nine fixing holes for camera positioning. **(C)** The mounting template.

**Figure 8:**
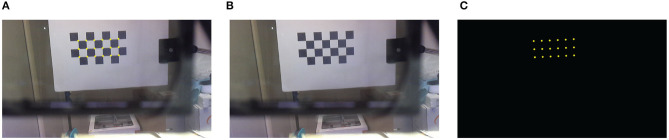
Viewpoint camera frames. **(A)** Camera frame of the augmented scene. **(B)** Camera frame of the real-world scene with display and optical shutter turned off. **(C)** Camera frame of the virtual scene with display and optical shutter turned on.

**Figure 9:**
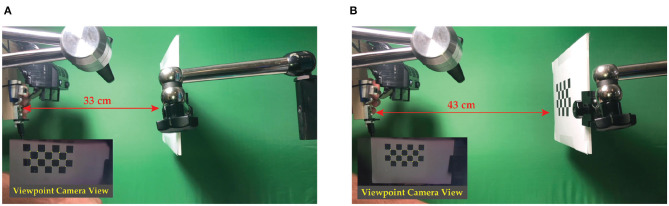
Images grabbed during two different testing sessions: in **(A)** the validation checkerboard is placed at 33 cm from the viewpoint camera, whereas in **(B)** the validation checkerboard is placed at 43 cm from the viewpoint camera. Bottom-left detail shows the viewpoint camera view of the augmented scene.

The validation checkerboard was placed at 16 different distances from the viewpoint camera, ranging from 18 to 65 cm (18 ≤ *d*^**C**→π^ ≤ 65 cm). Both the OST HMD and the validation checkerboard were locked by means of two rigid and adjustable holders. The viewpoint camera was attached to a 3D printed mounting template. The mounting template was equipped with fixing holes for placing the camera in eight different pre-set viewpoint positions radially arranged within the eye-box of the see-through display (Figure 7). Each viewpoint position was therefore at a distance of 4 mm from the calibration position (||(*x*′, *y*′)|| = 4 mm and *z* ′ ≈ 0 mm). The template and the camera were both anchored to the translation bar of the HMD holder. For each position of the viewpoint camera, and for each checkerboard position, a viewpoint camera image of the validation board was captured with the display and the optical shutter turned off (Figure 8B). Without moving the board or the camera, the set of virtual spots rendered by the OST display was then captured by the viewpoint camera with both the display and the optical shutter turned on in order to remove the real-world background (Figure 8C). The two images were processed separately by a user-guided semi-automatic corner detection algorithm. The pixel locations of the 18 inner corners of the checkerboard were used for the evaluation. The registration error was computed as the Euclidean distance between the virtual and real features (Figure 9).

### 5.2. Results

Table 2 shows the registration errors obtained for the viewpoint camera placed in the calibration position; the errors are grouped for the 16 clusters associated with the 16 positions where the validation checkerboard was placed (i.e., for 18 ≤ *d*^**C**→π^ ≤ 65 cm). In particular, the table reports the mean and standard deviation of respectively: the image registration error (pixel), the associated angular registration error (arcmin), and the absolute registration error (mm) measured by backprojecting the image registration error at the current chessboard distance (*d*^**C**→π^).

**Table 2:** Registration error measured at different viewpoint-to-checkerboard distances for the calibration position.

**Mean**	**Checkerboard distances (cm)**															
**(**σ**)**	**18–20**	**21–23**	**24–26**	**27–29**	**30–32**	**33–35**	**36–38**	**39–41**	**42–44**	**45–47**	**48–50**	**51–53**	**54–56**	**57–59**	**60–62**	**63–65**
Image	16.7	11.9	7.1	6.4	6.6	4.9	4.4	3.7	3.6	3.8	4	4.1	4.2	4.1	4.2	4.3
Registration error (px)	(11.9)	(7.9)	(3.9)	(2.7)	(2.8)	(1.9)	(1.9)	(1.5)	(1.2)	(1)	(0.7)	(0.8)	(0.8)	(0.8)	(0.7)	(0.6)
Angular	44.5	31.7	18.9	17.0	17.7	13.1	11.9	9.8	9.8	10.3	10.6	10.9	11.3	11.0	11.4	11.6
Registration error (arcmin)	(31.5)	(21.0)	(10.4)	(7.2)	(7.5)	(5.0)	(4.9)	(3.9)	(3.2)	(2.5)	(1.9)	(2.1)	(2.1)	(2.2)	(2.0)	(1.6)
Absolute	2.8	2	1.3	1.3	1.5	1.2	1.2	1	1.2	1.3	1.4	1.6	1.7	1.7	1.9	2
Registration error (mm)	(1.9)	(1.3)	(0.7)	(0.5)	(0.6)	(0.5)	(0.5)	(0.4)	(0.4)	(0.4)	(0.3)	(0.3)	(0.3)	(0.4)	(0.4)	(0.3)

Overall, the mean image registration error, angular registration error, and absolute registration for the calibration position ‖*E*_*c*_‖ were 5.87 px, 15.7 arcmin, and 1.57 mm. We can consider this contribution to the registration error as the intrinsic registration error of the system after the calibration, devoid of any parallax contribution due to the viewpoint shift. When analyzing the registration errors obtained for the other eight viewpoint positions, we reasonably considered the average registration error computed for the calibration position as the minimum error achievable. Results of the tests are reported in Table 3. The mean and standard deviation associated with each cluster of distances were computed over the 8 viewpoint positions.

**Table 3:** Registration error measured at different viewpoint-to-checkerboard distances for all the positions of the mounting template.

**Mean**	**Checkerboard distances (cm)**															
**(**σ**)**	**18–20**	**21–23**	**24–26**	**27–29**	**30–32**	**33–35**	**36–38**	**39–41**	**42–44**	**45–47**	**48–50**	**51–53**	**54–56**	**57–59**	**60–62**	**63–65**
Image	17.4	13.1	11.5	8.9	7.3	7	7.3	7	7.9	9.3	8.5	8.9	9	10.6	11.3	10.7
Registration error (px)	(8.3)	(7.3)	(5.9)	(3.7)	(3.3)	(2.2)	(2.6)	(3.2)	(3.3)	(2.3)	(4.2)	(4.4)	(5)	(4.7)	(4.8)	(5.1)
Angular	46.5	34.9	30.7	23.9	19.4	17.9	19.4	18.6	21.0	24.9	22.7	23.8	23.9	28.3	30.2	28.6
Registration error (arcmin)	(22.0)	(19.5)	(15.7)	(9.9)	(8.8)	(5.8)	(6.9)	(8.6)	(8.9)	(6.2)	(11.2)	(11.8)	(13.3)	(12.5)	(12.9)	(13.6)
Absolute	2.5	2.1	2.1	1.8	1.6	1.7	2	2	2.5	3.1	3	3.4	3.6	4.5	5	4.9
Registration error (mm)	(1.2)	(1.1)	(1.1)	(0.7)	(0.7)	(0.6)	(0.7)	(0.9)	(1)	(0.8)	(1.5)	(1.7)	(1.9)	(2)	(2.1)	(2.4)

By means of Equation (22), we can model the trend of the magnitude of the registration error (||*E*||) as a function of the depth (i.e., the distance of the plane under observation ${\text{p}}_{z}^{{\text{R}}_{\text{C}}}$). To a first approximation, we can reasonably ignore the contribution to the registration error due to the *z*′ component of the viewpoint shift, for two reasons:

- In our setup, the viewpoint camera is forced to shift on a plane approximately parallel to the image plane of the display, namely *z*′ ≈ 0
- The ratio $\frac{{\text{p}}_{x}^{{\text{R}}_{\text{C}}}}{{\text{p}}_{z}^{{\text{R}}_{\text{C}}}}$ (x and z coordinate of a 3D point in the off-axis rendering camera) is at most equal to *tan*(*hfov*/2) ≈ 0.21, since the evaluation of the registration error can be done only for those points in space that are within the horizontal fov of the OST display. The same consideration applies for $\frac{{\text{p}}_{y}^{{\text{R}}_{\text{C}}}}{{\text{p}}_{z}^{{\text{R}}_{\text{C}}}}$ that is at most equal to *tan*(*vfov*/2) ≈ 0.17.

Therefore, the simplified version of the magnitude of the vector of registration error from 22 is:


(17) $$\begin{array}{l} ||\text{E}||=||\frac{{\text{p}}_{z}^{{\text{R}}_{\text{C}}}+z^{\prime }}{f}\left({\left({\text{i}}^{\text{S}}\right)}_{\text{V}}-{\left({\text{i}}^{\text{S}}\right)}_{\text{C}}\right)||\approx \|\begin{array}{c} -x^{\prime }\left(\frac{{\text{p}}_{z}^{{\text{R}}_{\text{C}}}}{d^{\text{C}\to \pi }}-1\right)\\ -y^{\prime }\left(\frac{{\text{p}}_{z}^{{\text{R}}_{\text{C}}}}{d^{\text{C}\to \pi }}-1\right) \end{array}\|\\ \;=O^{\prime }||\left(\frac{{\text{p}}_{z}^{{\text{R}}_{\text{C}}}}{d^{\text{C}\to \pi }}-1\right)|| \end{array}$$

where *O*′ is the radial viewpoint parallax (in our tests we have *O*′ = 4 mm).

**Figure 10:**
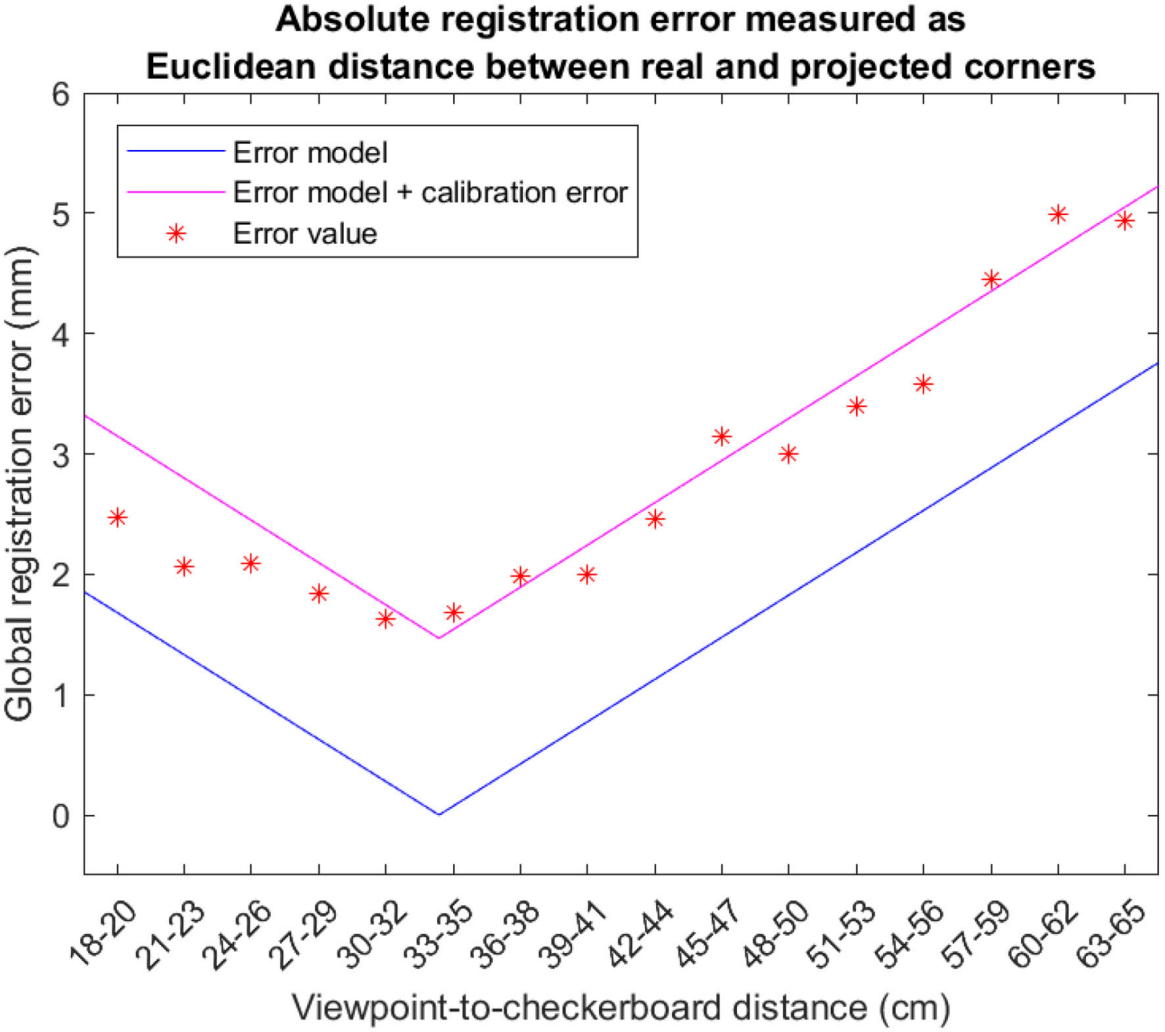
Absolute registration error for the 16 clusters associated with the 16 positions where the validation checkerboard was placed (i.e., for 18 ≤ *d*^**C**→π^ ≤ 65 cm). Each asterisk is the average of the absolute registration error computed over the eight viewpoint positions. The lines represent the theoretical registration error due to the viewpoint shift as calculated through the Equation (23). As predicted by our model, the minimum of the curve representing the experimental tests is extremely close to the value of the display focal distance (i.e., ≈ 33 cm).

Figure 10 reports the trend of the absolute registration error measured at the various checkerboard distances. For each cluster, the asterisks represent the mean error over the 8 viewpoint positions, the black line represents the theoretical magnitude of the registration error due to the viewpoint shift as calculated through Equation (23), and the pink line represents the theoretical magnitude of the registration error shifted by a factor that corresponds to the contribution of the calibration error (||*E*|| + ||*E*_*c*_||).

## 6. Discussion

As shown in Figure 10, for checkerboard distances ${\text{p}}_{z}^{{\text{R}}_{\text{C}}}>d^{\text{C}\to \pi }$ and ${\text{p}}_{z}^{{\text{R}}_{\text{C}}}<d^{\text{C}\to \pi }$, taking into account all the 8 viewpoint camera positions associated with a parallax of magnitude *O*′, the estimated absolute registration error increases. This increment is properly modeled by Equation (23) accounting for the contribution of the viewpoint shift to the overall registration error.

The results obtained with the experimental tests confirm rather accurately the trend of the theoretical model represented by Equation (23) and comprising also the contribution of the calibration error (||*E*|| + ||*E*_*c*_||). It should be also noted that, as predicted by our model, the minimum of the curve representing the experimental tests is extremely close to the value of the display focal distance (i.e., ≈ 33 cm).

The results reported in Tables 2, 3 show that, for a reasonably wide range of distances from the focal plane of the OST display (i.e., $21<{\text{p}}_{z}^{{\text{R}}_{\text{C}}}<41$ cm), the mean absolute registration error due to a viewpoint shift of magnitude 4 mm is comparable to that obtained for the calibration viewpoint: to a first approximation, given a viewpoint shift of 4 mm with respect to the calibration point, the parallax contribution to the registration error is ≤ 0.6 mm for target objects placed at $21<{\text{p}}_{z}^{{\text{R}}_{\text{C}}}<41$ cm (1.3 mm of mean absolute registration error for the calibration position vs. 1.9 mm for the shifted viewpoint positions). This absolute registration error is reasonably low to be considered as sufficiently reliable to guide high-precision manual procedures.

We hypothesize that the non-negligible magnitude of ||*E*_*c*_|| is due to inaccuracies in the calibration. By means of example, in our calibration procedure, we did not consider the non-linear distortions due to the optics of the display, whereas a certain amount of image distortion (such as the radial distortion) is certainly present. As suggested in Lee and Hua (2015), a camera-based calibration method that tackled this problem and estimates also the non-linearities in the projection model of the OST display due to the optical distortions would likely provide better results in terms of registration accuracy.

The global registration error trend is slightly different from the theoretical parallax error contribution. This discrepancy could be due to the non-perfectly accurate estimation of the rotation matrix ${\;}_{\text{ C}}^{{\text{R}}_{\text{D}}}\text{R}$ performed during the calibration procedure. As illustrated in section 4, this rotational contribution is used to estimate the orientation of the tracking camera with respect to the rendering camera of the display ${\;}_{\text{L}}^{{\text{R}}_{\text{D}}}\text{R}$. In order to improve the accuracy of this part of the calibration procedure, we could measure the final calibration result of ${\;}_{\text{t}}^{\text{R}}\text{R}$ by averaging from a set of repeated measurements obtained by placing the viewpoint camera in different calibration positions and calculating, for each position, ${\;}_{\text{ C}}^{{\text{R}}_{\text{D}}}\text{R}{\;}_{\text{L}}^{\text{C}}\text{R}$. To this aim, a more elaborated stereo calibration procedure, based on global calibration technique capable to counter some of the causes of calibration errors could improve the overall calibration accuracy (Chen et al., 2019). In the future, we are planning to carry out a more detailed error analysis on the possible sources of inaccuracies in the stereo-calibration.

In order to provide a preliminary evaluation of the proposed solution with human subjects, we have performed a preliminary user study: six lab members, including the authors, were asked to monocularly look at the right display of the HMD to judge the virtual-to-real registration accuracy obtained while observing a checkerboard placed at approximately 33 cm. All of the subjects evaluated that the alignment was accurate at a first glance. Nevertheless, more objective tests are still needed in order to robustly assess our solution also in terms of vergence-accommodation conflict and focus rivalry, as these specific perceptual aspects were not considered in the paper.

Better results could be also attained during these user tests if we a prior-to-use quick SPAAM-like manual calibration refinement was performed to roughly estimate the viewpoint position and therefore to reduce the magnitude of *O*′. This approach would be similar to the official Microsoft HoloLens 1 calibration procedure that is used to approximately estimate the user's interpupillary distance (Grubert et al., 2018). Finally, the possibility to estimate the registration error due to the viewpoint shift offered by our model would allow the user to be notified, in a real application, whenever the AR view cannot ensure that the AR registration fall within a certain margin of accuracy.

## 7. Conclusion and Future Work

In literature, the idea of adjusting the optical depth of the virtual image to match the depth of the fixation point of the user was primarily explored with the aim to reduce the vergence-accommodation conflict inherent to near-eye-displays used in the peripersonal space (Dunn et al., 2018). In this paper, we demonstrated the beneficial impact, on the virtual-to-real registration, of the use of AR OST displays with optical engines that collimate the computer-generated image at a depth that matches the fixation point of the user in the peripersonal space. This strategy is based on the use of displays with short focal distances and it features a dedicated parameterization of the virtual rendering camera based on an automatic calibration routine to estimate the projection parameters of the OST display for a generic viewpoint position.

In the work, we have first built a theoretical model of the registration error and then we have experimentally proved its ability to predict the registration error, due to a specific viewpoint shift, outside the distance of the focal plane of the display. We have also demonstrated that, with this solution, there is reasonably no need for any prior-to-use calibration refinement, either manual or interaction-free, to ensure the accurate virtual-to-real registration provided that the view volume under observation stays within a suitable depth range around the display focal plane. This finding will pave the way to the development of new multi-focal models of OST HMDs specifically conceived as aid during high-precision manual tasks in the peripersonal space.

To counter some of the limitations of our validation tests, future work will involve improving the calibration procedure in order to estimate also the radial and tangential distortions caused by the collimation optics of the display, and to estimate more accurately the orientation of the tracking sensor/camera with respect to the display. In addition, we plan to conduct a rigorous validation of the proposed method by separating the contribution to the registration error due to the viewpoint shift from the contribution due to the tracking inaccuracies. Finally, future work will also include experimental tests involving actual users, and based on the manipulation of three-dimensional objects in the peripersonal space. With this user study, we will be able also to evaluate the efficacy of the proposed strategy in mitigating the vergence-accommodation conflict and the focus rivalry problem typical of OST HMDs.

## Data Availability Statement

The original contributions presented in the study are included in the article/Supplementary Materials, further inquiries can be directed to the corresponding author/s.

## Author Contributions

FC, VF, and NC conceived of the presented idea. FC developed the theory and performed the computations. NC, UF, and FC implemented the software. NC and UF carried out the experiments. FC, NC, and VF verified the analytical methods. FC wrote the manuscript. NC took care of the writing review and editing. FC and VF were in charge of overall direction and planning. VF supervised the project. All authors discussed the results, and have read and agreed to the published version of the manuscript.

## Conflict of Interest

The authors declare that the research was conducted in the absence of any commercial or financial relationships that could be construed as a potential conflict of interest.

## References

[B1] Cattari N. Cutolo F. D'amato R. Fontana U. Ferrari V. (2019). Toed-in vs parallel displays in video see-through head-mounted displays for close-up view. IEEE Access 7, 159698–159711. 10.1109/ACCESS.2019.2950877

[B2] Chen M. Tang Y. Zou X. Huang K. Li L. He Y. (2019). High-accuracy multi-camera reconstruction enhanced by adaptive point cloud correction algorithm. Opt. Lasers Eng. 122, 170–183. 10.1016/j.optlaseng.2019.06.011

[B3] Condino S. Turini G. Parchi P. D. Viglialoro R. M. Piolanti N. Gesi M.. (2018). How to build a patient-specific hybrid simulator for orthopaedic open surgery: Benefits and limits of mixed-reality using the microsoft hololens. J. Healthcare Eng. 2018:5435097. 10.1155/2018/5435097 30515284 PMC6236521

[B4] Cutolo F. (2019). Letter to the editor on “augmented reality based navigation for computer assisted hip resurfacing: a proof of concept study”. Ann. Biomed. Eng. 47, 2151–2153. 10.1007/s10439-019-02299-w 31161302

[B5] Cutolo F. Fida B. Cattari N. Ferrari V. (2020). Software framework for customized augmented reality headsets in medicine. IEEE Access 8, 706–720. 10.1109/ACCESS.2019.2962122

[B6] Cutolo F. Fontana U. Carbone M. D'Amato R. Ferrari V. (2017). “Hybrid video/optical see-through HMD,” in 2017 IEEE International Symposium on Mixed and Augmented Reality (ISMAR-Adjunct) (Nantes), 52–57.

[B7] Cutolo F. Fontana U. Cattari N. Ferrari V. (2019). Off-line camera-based calibration for optical see-through head-mounted displays. Appl. Sci. 10:193. 10.3390/app10010193

[B8] Dunn D. Chakravarthula P. Dong Q. Fuchs H. (2018). “Mitigating vergence-accommodation conflict for near-eye displays via deformable beamsplitters,” in Digital Optics for Immersive Displays, eds B. C. Kress, W. Osten, and H. Stolle (Strasbourg: International Society for Optics and Photonics, SPIE), 196–208.

[B9] Ferrari V. Cattari N. Fontana U. Cutolo F. (2020). Parallax free registration for augmented reality optical see-through displays in the peripersonal space. IEEE Trans. Visual. Comput. Graph. 10.1109/TVCG.2020.3021534 32881688

[B10] Genc Y. Tuceryan M. Navab N. (2002). “Practical solutions for calibration of optical see-through devices,” in Proceedings of the IEEE and ACM International Symposium on Mixed and Augmented Reality (ISMAR 2002) (Darmstadt), 169–175.

[B11] Gilson S. J. Fitzgibbon A. W. Glennerster A. (2008). Spatial calibration of an optical see-through head-mounted display. J. Neurosci. Methods 173, 140–146. 10.1016/j.jneumeth.2008.05.015 18599125 PMC2816817

[B12] Grubert J. Itoh Y. Moser K. Swan J. E. (2018). A survey of calibration methods for optical see-through head-mounted displays. IEEE Trans. Visual. Comput. Graph. 24, 2649–2662. 10.1109/TVCG.2017.2754257 28961115

[B13] Guestrin E. D. Eizenman M. (2006). General theory of remote gaze estimation using the pupil center and corneal reflections. IEEE Trans. Biomed. Eng. 53, 1124–1133. 10.1109/TBME.2005.863952 16761839

[B14] Holliman N. S. Dodgson N. A. Favalora G. E. Pockett L. (2011). Three-dimensional displays: a review and applications analysis. IEEE Trans. Broadcast. 57, 362–371. 10.1109/TBC.2011.2130930

[B15] Itoh Y. Klinker G. (2014a). “Interaction-free calibration for optical see-through head-mounted displays based on 3D eye localization,” in 2014 IEEE Symposium on 3D User Interfaces (3DUI) (Minneapolis, MN: IEEE), 75–82.

[B16] Itoh Y. Klinker G. (2014b). “Performance and sensitivity analysis of INDICA: INteraction-free DIsplay CAlibration for optical see-through head-mounted displays,” in 2014 IEEE International Symposium on Mixed and Augmented Reality (ISMAR) (IEEE), 171–176.

[B17] Lee S. Hua H. (2015). A robust camera-based method for optical distortion calibration of head-mounted displays. J. Display Technol. 11, 845–853. 10.1109/JDT.2014.2386216

[B18] Luo G. Rensing N. M. Weststrate E. Peli E. (2005). Registration of an on-axis see-through head-mounted display and camera system. Opt. Eng. 44, 1–7. 10.1117/1.1839231

[B19] Moser K. R. Swan J. E. (2016). “Evaluation of user-centric optical see-through head-mounted display calibration using a leap motion controller,” in 2016 IEEE Symposium on 3D User Interfaces (3DUI) (Greenville, SC), 159–167.

[B20] Navab N. Zokai S. Genc Y. Coelho E. M. (2004). “An on-line evaluation system for optical see-through augmented reality,” in In Proceedings of the IEEE Virtual Reality 2004 (IEEE VR 2004) (Chicago, IL), 245–246.

[B21] Owen C. B. Zhou J. Tang A. Xiao F. (2004). “Display-relative calibration for optical see-through head-mounted displays,” in Third IEEE and ACM International Symposium on Mixed and Augmented Reality (Arlington, VA: IEEE), 70–78.

[B22] Plopski A. Itoh Y. Nitschke C. Kiyokawa K. Klinker G. Takemura H. (2015). Corneal-imaging calibration for optical see-through head-mounted displays. IEEE Trans. Visual. Computer Graph. 21, 481–490. 10.1109/TVCG.2015.2391857 26357098

[B23] Qian L. Azimi E. Kazanzides P. Navab N. (2017a). Comprehensive tracker based display calibration for holographic optical see-through head-mounted display. arXiv preprint arXiv:1703.05834.

[B24] Qian L. Barthel A. Johnson A. Osgood G. Kazanzides P. Navab N.. (2017b). Comparison of optical see-through head-mounted displays for surgical interventions with object-anchored 2D-display. Int. J. Comput. Assist. Radiolo. Surg. 12, 901–910. 10.1007/s11548-017-1564-y PMC5891507 28343301

[B25] Rolland J. P. Cakmakci O. (2005). “The past, present, and future of head-mounted display designs,” in Optical Design and Testing II, eds Y. Wang, Z. Weng, S. Ye, and J. M. Sasian (Beijing: International Society for Optics and Photonics, SPIE) 368–377.

[B26] Rolland J. P. Fuchs H. (2000). Optical versus video see-through head-mounted displays in medical visualization. Presence Teleoperat. Virt. Environ. 9, 287–309. 10.1162/105474600566808

[B27] Sielhorst T. Bichlmeier C. Heining S. M. Navab N. (2006). “Depth perception – a major issue in medical ar: Evaluation study by twenty surgeons,” in Medical Image Computing and Computer-Assisted Intervention – MICCAI 2006, eds R. Larsen, M. Nielsen, and J. Sporring (Berlin; Heidelberg: Springer), 364–372. 10.1007/11866565_45 17354911

[B28] Tuceryan M. Genc Y. Navab N. (2002). Single-point active alignment method (SPAAM) for optical see-through HMD calibration for augmented reality. Presence Teleoper. Virt. Environ. 11, 259–276. 10.1162/105474602317473213

[B29] van Krevelen D. W. F Poelman R. (2010). A survey of augmented reality technologies, applications and limitations. Int. J. Virt. Real. 9, 1–20. 10.20870/IJVR.2010.9.2.2767

[B30] Vávra P. Roman J. Zonča P. P.Ihnát Němec, M. Kumar J.. (2017). Recent development of augmented reality in surgery: A review. J. Healthcare Eng. 2017:4574172. 10.1155/2017/4574172 PMC5585624 29065604

[B31] Zhang Z. (2000). A flexible new technique for camera calibration. IEEE Trans. Pattern Anal. Mach. Intell. 22, 1330–1334. 10.1109/34.888718a

