# Corrigendum: Optical See-Through Head-Mounted Displays With Short Focal Distance: Conditions for Mitigating Parallax-Related Registration Error

**DOI:** 10.3389/frobt.2022.930382

**Published:** 2022-06-07

**Authors:** Fabrizio Cutolo, Nadia Cattari, Umberto Fontana, Vincenzo Ferrari

**Affiliations:** ^1^ Information Engineering Department, University of Pisa, Pisa, Italy; ^2^ Department of Translational Research and New Technologies in Medicine and Surgery, EndoCAS Center, University of Pisa, Pisa, Italy

**Keywords:** augmented reality, optical see-through displays, registration, calibration, parallax related error

In the original article, there was a mistake in the content of Tables 2 and 3 as published. The values of the mean and standard deviation of the virtual-to-real overlay error in visual angles, which are reported in the third and fourth rows of Tables 2 and 3, are to be corrected. Due to a typing error within the data analysis code, we mistakenly considered an erroneous value of the average angular resolution for the eye-replacement camera. This scale factor is used to pass from the original registration errors (expressed in pixel) to the angular registration errors (in arcmin). The value of the average angular resolution is ≈2.67 arcmin/pixel. The corrected Tables 2 and 3 appear below.

**TABLE 2:** Registration error measured at different viewpoint-to-checkerboard distances for the calibration position.

Mean	Checkerboard distances (cm)															
(*σ*)	18–20	21–23	24–26	27–29	30–32	33–35	36–38	39–41	42–44	45–47	48–50	51–53	54–56	57–59	60–62	63–65
Image	16.7	11.9	7.1	6.4	6.6	4.9	4.4	3.7	3.6	3.8	4	4.1	4.2	4.1	4.2	4.3
Registration error (px)	(11.9)	(7.9)	(3.9)	(2.7)	(2.8)	(1.9)	(1.9)	(1.5)	(1.2)	(1)	(0.7)	(0.8)	(0.8)	(0.8)	(0.7)	(0.6)
Angular	44.5	31.7	18.9	17.0	17.7	13.1	11.9	9.8	9.8	10.3	10.6	10.9	11.3	11.0	11.4	11.6
Registration error (arcmin)	(31.5)	(21.0)	(10.4)	(7.2)	(7.5)	(5.0)	(4.9)	(3.9)	(3.2)	(2.5)	(1.9)	(2.1)	(2.1)	(2.2)	(2.0)	(1.6)
Absolute	2.8	2	1.3	1.3	1.5	1.2	1.2	1	1.2	1.3	1.4	1.6	1.7	1.7	1.9	2
Registration error (mm)	(1.9)	(1.3)	(0.7)	(0.5)	(0.6)	(0.5)	(0.5)	(0.4)	(0.4)	(0.4)	(0.3)	(0.3)	(0.3)	(0.4)	(0.4)	(0.3)

**TABLE 3:** Registration error measured at different viewpoint-to-checkerboard distances for all the positions of the mounting template.

Mean	Checkerboard distances (cm)															
(*σ*)	18–20	21–23	24–26	27–29	30–32	33–35	36–38	39–41	42–44	45–47	48–50	51–53	54–56	57–59	60–62	63–65
Image	17.4	13.1	11.5	8.9	7.3	7	7.3	7	7.9	9.3	8.5	8.9	9	10.6	11.3	10.7
Registration error (px)	(8.3)	(7.3)	(5.9)	(3.7)	(3.3)	(2.2)	(2.6)	(3.2)	(3.3)	(2.3)	(4.2)	(4.4)	(5)	(4.7)	(4.8)	(5.1)
Angular	46.5	34.9	30.7	23.9	19.4	17.9	19.4	18.6	21.0	24.9	22.7	23.8	23.9	28.3	30.2	28.6
Registration error (arcmin)	(22.0)	(19.5)	(15.7)	(9.9)	(8.8)	(5.8)	(6.9)	(8.6)	(8.9)	(6.2)	(11.2)	(11.8)	(13.3)	(12.5)	(12.9)	(13.6)
Absolute	2.5	2.1	2.1	1.8	1.6	1.7	2	2	2.5	3.1	3	3.4	3.6	4.5	5	4.9
Registration error (mm)	(1.2)	(1.1)	(1.1)	(0.7)	(0.7)	(0.6)	(0.7)	(0.9)	(1)	(0.8)	(1.5)	(1.7)	(1.9)	(2)	(2.1)	(2.4)

For the same reason, in the original article, there was an error at page 10 in the text where we reported the overall mean angular registration error. As previously stated, we considered an erroneous scale factor to convert the image registration error in pixel to the angular registration error. A correction has been made to **Section 5 “Experiments and Results,”** *Section 5.2 “Results”:*

“Overall, the mean image registration error, angular registration error, and absolute registration for the calibration position ‖E_c_‖ were 5.87 px, 15.7 arcmin, and 1.57 mm.”

The authors apologize for these errors and state that these do not change the scientific conclusions of the article in any way. The original article has been updated.

